# The Effects of Old Age Care Services and Long-Term Care Insurance: Evidence From Korea

**DOI:** 10.1093/geroni/igaa057.281

**Published:** 2020-12-16

**Authors:** Hoolda Kim, Sophie Mitra

**Affiliations:** 1 Black Hills State University, Spearfish, South Dakota, United States; 2 Fordham University, Bronx, New York, United States

## Abstract

Along with a rapidly aging population globally, the need for homecare is on the rise. Such care can be a heavy financial burden on older people and their households. To address this issue, the Korean government initiated two programs: care services for low-income individuals and voluntary long-term care insurance for people age 65+. Although it has been a decade since the implementation of the programs, there is limited evidence on how they affect the economic lives of older people, with and without disabilities. We use the 2008-2018 Korean Longitudinal Study of Aging (KLoSa) that provides detailed information about healthcare and homecare utilization and out of pocket costs for people aged 45 or older. We investigate the economic effects of old age public assistance programs using multivariate linear regression models and a dynamic panel model. The preliminary results indicate that older people with disabilities are more likely to utilize homecare services but do not experience any significant extra costs associated with homecare, implying that old age public assistance programs mitigate the financial burden of homecare for older people.

